# Evaluation of the Reliability of Electrocardiographic Criteria for Cardiac Hypertrophy Based on Echocardiographic Data

**DOI:** 10.15388/Amed.2021.29.1.12

**Published:** 2022-07-26

**Authors:** Agnė Augustaitytė, Eglė Kalinauskienė

**Affiliations:** Faculty of Medicine, Lithuanian University of Health Sciences, Kaunas, Lithuania; Faculty of Medicine, Lithuanian University of Health Sciences, Kaunas, Lithuania; Hospital of the Lithuanian University of Health Sciences Kauno klinikos, Kaunas, Lithuania

**Keywords:** ECG criteria, left ventricular hypertrophy, transthoracic echocardiography

## Abstract

**Background.:**

Left ventricular hypertrophy (LVH) regardless of other risk factors may be associated with an increased risk of mortality from cardiovascular diseases. Therefore, timely diagnosis for LVH is important in order to avoid possible complications. One of the simplest and cheapest methods to diagnose LVH is electrocardiography (ECG). Although a number of ECG criteria for LVH is known, their reliability varies in many studies.

**Aim.:**

To evaluate the reliability of ECG criteria for LVH based on transthoracic echocardiography (TTE) data.

**Methods.:**

The study included all consecutive patients in Kaunas Clinical Hospital Department of Cardiology from December 2019 until March 2020 and from September until October 2020, after applying the inclusion and exclusion criteria. The sensitivity and specificity of the ECG criteria for LVH were assessed based on TTE measurements performed during the same inpatient setting. The reliability of the ECG criteria for LVH was assessed using ROC curves. Reliability differences in gender, age and nutritional status groups were assessed using ANOVA statistical method.

**Results.:**

Data from 95 patients were analyzed (63.2% were women and 36.8% were men). The sensitivity, specificity and AUC of Sokolow–Lyon criterion were 9.38%, 85.71% and 0.44 (p = 0.034), R in aVL – 6.25%, 90.48% and 0.51 (p = 0.038), Cornell – 21.88%, 100 % and0.69 (p = 0.084), Cornell product – 31.25%, 95.24% and 0.72 (p = 0.070), Peguero–Lo Presti – 31.25%, 85.71% and 0.68 (p = 0.053), respectively. No statistically significant differences were observed among the individual gender, age and nutritional status groups.

**Conclusions.:**

Sokolow–Lyon and RaVL criteria were not statistically significantly reliable in LVH diagnosis compared to TTE, unlike the Cornell, Cornell product, and Peguero–Lo Presti criteria.

## Santrauka.

**Įvadas.:**

Kairiojo skilvelio hipertrofija (KSH) nepriklausomai nuo kitų rizikos veiksnių gali lemti padidėjusią mirtingumo nuo širdies ir kraujagyslių ligų riziką, todėl labai svarbi laiku atlikta KSH diagnostika siekiant išvengti galimų komplikacijų. Vienas iš paprasčiausių ir pigiausių metodų KSH nustatyti – elektrokardiografija (EKG). Nepaisant to, kad yra nemažai žinomų EKG kriterijų KSH nustatyti, jų patikimumas įvairiuose moksliniuose tyrimuose skiriasi.

**Tikslas.:**

Įvertinti elektrokardiografinių širdies KSH kriterijų patikimumą, remiantis transtorakalinės echokardiografijos (TTE) duomenimis.

**Metodai.:**

Į tyrimą įtraukti visi iš eilės pacientai, stacionarizuoti Kauno klinikinės ligoninės Kardiologijos skyriuje 2019 m. gruodžio mėn. – 2020 m. kovo mėn. ir 2020 m. rugsėjo – spalio mėn., pritaikius įtraukimo ir atmetimo kriterijus. EKG kriterijų jautrumas ir specifiškumas širdies KSH nustatyti vertintas, remiantis to paties stacionarizavimo metu atliktais TTE matavimais. EKG kriterijų patikimumas KSH diagnostikoje vertintas naudojant ROC kreives. ANOVA statistiniu metodu vertinti patikimumo skirtumai lyties, amžiaus ir mitybos būklės grupėse.

**Rezultatai.:**

Analizuoti 95 pacientų duomenys, iš kurių 63,2 % sudarė moterys ir 36,8 % – vyrai. Sokolow-Lyon kriterijaus jautrumas, specifiškumas ir AUC atitinkamai buvo 9,38 %, 85,71 % ir 0,44 (p = 0,034), RaVL – 6,25 %, 90,48 % ir 0,51 (p = 0,038), Cornell – 21,88 %, 100 % ir 0,69 (p = 0,084), Cornell produkto – 31,25 %, 95,24 % ir 0,72 (p = 0,070), Peguero-Lo Presti – 31,25 %, 85,71 % ir 0,68 (p = 0,053). Statistiškai reikšmingų skirtumų tarp atskirų lyties, amžiaus ir mitybos būklės grupių nebuvo nustatyta.

**Išvados.:**

Sokolow-Lyon ir RaVL kriterijai nebuvo statistiškai reikšmingai patikimi lyginant su TTE širdies KSH diagnostikoje, skirtingai nei Cornell, Cornell produkto bei Peguero-Lo Presti kriterijai.

## Introduction

According to World Health Organization (WHO) data from year 2017, deaths from cardiovascular diseases account for about a third of all deaths every year [1]. Regardless of other factors, left ventricular hypertrophy (LVH) is associated with an increased risk of mortality from cardiovascular diseases. As a clinical study in Korea showed, myocardial mass index (MMI) is directly correlated with QRS voltage [2]. Therefore, the diagnosis of LVH is currently based on electrocardiography (ECG) as well as on transthoracic echocardiography (TTE). However, it has been observed that the sensitivity and specificity of ECG criteria for LVH are not as high as using TTE or other diagnostic methods [3]. The aim of this study is to review not only those ECG criteria that are already used in medical practice but also newly described in the literature and to determine which of them are the most reliable indicators for LVH.

## Methods

A prospective study was performed that included all consecutive patients admitted to the Cardiology Department of Kaunas Clinical Hospital (KCH) from December 2019 until October 2020, excluding the period of quarantine for coronavirus disease 2019 (COVID-19) (from April 2020 until August 2020). Inclusion and exclusion criteria for entry were applied. Inclusion criteria: patient above 18 years of age, written consent for participation in the study, obtaining ECG recording and a scheduled TTE. Exclusion criteria: paced rhythm in ECG recording (except atrial only), preexitation, right or left bundle branch block.

General data (age, gender, height, weight) and ECGs were collected from patients’ medical charts. In this study, the above-mentioned general data parameters are reported as mean ± standard deviation. According to the WHO classification, the subjects were divided into 2 groups based on age: young patients (< 65 years of age) and elderly (≥ 65 years of age) [4]. Patients were divided into the following nutritional status groups according to their body mass index (BMI): normal body mass index (18.5–24.9 kg/m^2^), preobesity (25.0–29.9 kg/m^2^), obesity class I (30.0–34.9 kg/m^2^), obesity class II (35.0–39.9 kg/m^2^), obesity class III (≥ 40.0 kg/m^2^) [5].

ECG analysis was performed manually by a single person. The following LVH criteria were assessed:

1) Sokolow–Lyon: SV_1_ + RV_5_ or SV_1_ + RV_6_ ≥ 35 mm.
2) RaVL: R wave amplitude (mm) in lead aVL ≥ 11 mm.
3) Cornell voltage: SV_3_ + RaVL > 28 mm for men and > 20 mm for women.
4) Cornell product: (SV_3_ + RaVL + 6) x QRS duration for women and (SV_3_ + RaVL) x QRS duration for men > 2440 mm x ms.
5) Peguero–Lo Presti: S_deepest_ + SV_4_ > 28 mm for men and > 23 mm for women, where S_deepest_ is the deepest S wave in any lead.

During the same hospitalization, TTE data were collected from the same patients’ medical charts: MMI, relative wall thickness (RWT), interventricular septal thickness (IVSd), left ventricular posterior wall thickness at end-diastole (PWTd). Echocardiography was performed by the cardiologists who worked in the department. IVSd, left ventricular internal diameter at end-diastole (LVIDd), and PWTd were measured manually. The remaining parameters – left ventricular myocardial mass (LVMM), MMI, and RWT – were automatically calculated by echocardiograph [3].

According to the European Association for Cardiovascular Imaging (EACVI), normal values of IVSd were considered to be 6–9 mm for women and 6–10 mm for men, and normal values of PWTd for women and men were considered to be 6–9 mm and 6–10 mm, respectively [3]. Based on MMI and RWT four LV structure groups were formed:

1) Normal LV structure: MMI 49–115 g/m^2^ for men or 43–95 g/m^2^ for women with RWT ≤ 0.42.
2) Concentric LVH: MMI > 115 g/m^2^ for men or > 95 g/m^2^ for women with RWT > 0.42.
3) Eccentric LVH: MMI > 115 g/m^2^ for men or > 95 g/m^2^ for women with RWT ≤ 0.42.
4) LV remodeling: MMI 49–115 g/m^2^ for men or 43–95 g/m^2^ for women with RWT > 0.42.

The sensitivity and specificity of the ECG criteria were calculated and compared, as well as ROC curves were drawn to determine the reliability of the ECG criteria for the diagnosis of LVH:


$$\mathrm{Sensitivity}\;=\frac{\mathrm{True}\;\mathrm{positive}\;\mathrm{values}}{\mathrm{True}\;\mathrm{positive}\;\mathrm{values}\;+\;\mathrm{False}\;\mathrm{negative}\;\mathrm{values}}\times 100\%$$

$$\mathrm{Specificity}\;=\frac{\mathrm{True}\;\mathrm{negative}\;\mathrm{values}}{\mathrm{True}\;\mathrm{negative}\;\mathrm{values}\;+\;\mathrm{False}\;\mathrm{positive}\;\mathrm{values}}\times 100\%$$

Using ROC curves, the area under the curve (AUC) of each ECG criterion was calculated. The sensitivity and specificity of TTE were assumed to be 100% (AUC = 1). Using Chi-Square test, the reference AUC = 1 was compared to each criterion’s AUC. In this way, the hypothesis that the reliability of the diagnostic ECG criterion did not differ from the reliability of TTE was tested. A 95% confidence interval (CI) was chosen, meaning that at p < 0.05, the reliability of ECG criterion was considered to be statistically significantly different from the TTE reliability.

The reliabilities of the examined ECG criteria in different gender, nutritional status and age groups were calculated as well. The reliability of Cornell voltage, Cornell product and Peguero–Lo Presti criteria has not been assessed in terms of gender, as the Cornell voltage and Peguero–Lo Presti criteria already have separate thresholds depending on gender, and the Cornell product criterion is calculated differently for men and women. The reliability of the examined ECG criteria was calculated using generalized linear model. Analysis of variance (ANOVA) statistical method was used to test the hypothesis, whether there was a statistically significant difference between all the data of each ECG criterion and the data segregated by gender, nutritional status, and age groups. The hypothesis was accepted at p < 0.05 (statistically significant difference between groups) and rejected at p > 0.05.

Sensitivity, specificity and other calculations of ECG criteria were performed using Microsoft Excel 2019 and R-Statistics programs. Graphs were made using the R-Statistics program.

## Results

### General parameters

There were 95 participants in the study that met the study criteria. A total of 60 (63.2%) women and 35 (36.8%) men were included in the study. Patients had a mean age of 71.2 ± 11.4 years, with minimum and maximum age of 48 and 90 years, respectively. Patients aged < 65 years accounted for 29.5% and those ≥ 65 years old accounted for 70.5% of all subjects.

The mean height and weight of the patients were 1.67 ± 0.11 m and 87.27 ± 20.40 kg, respectively. The mean BMI was 31.0 ± 6.2 kg / m^2^. Based on BMI, patients were divided into nutritional status groups (Table 1). Patients with I–III class of obesity (49.5%) and preobesity (37.9%) accounted for the largest proportion of subjects, with a minority (12.6%) having a normal BMI.

**Table 1.** Nutritional status groups based on BMI

Nutritional status group	Subjects (%)
Normal body mass index	12.6
Preobesity	37.9
Obesity class I	31.6
Obesity class II	10.5
Obesity class III	7.4

### Values of ECG criteria

The analyzed ECG criteria for LVH were met only in a small portion of the registered ECGs. Nevertheless, Cornell product and Peguero–Lo Presti criteria were met most frequently (13.7% and 17.9%, respectively). The percentage distribution of met and unmet ECG criteria is shown in Table 2.

**Table 2.** The percentage distribution of met and unmet ECG criteria

ECG criterion	Met criterion (%)	Unmet criterion (%)
Sokolow–Lyon	7.4	92.6
RaVL	6.3	93.7
Cornell voltage	8.4	91.6
Cornell product	13.7	86.3
Peguero–Lo Presti	17.9	82.1

### TTE data

**Figure 1.**
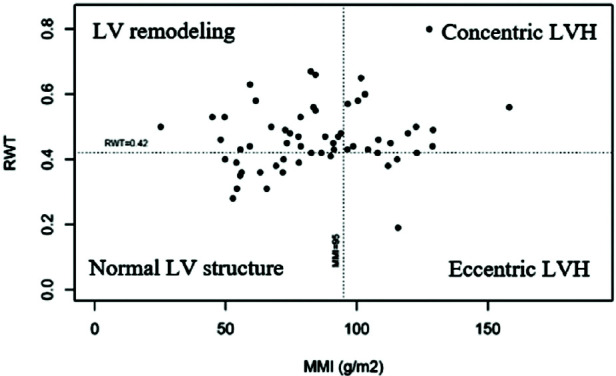
TTE data distribution among women (LV – left ventricle; LVH – left ventricular hypertrophy; RWT – relative wall thickness; MMI – myocardial mass index)

**Figure 2.**
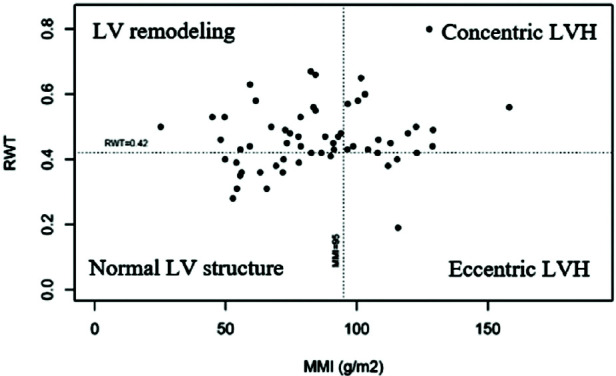
TTE data distribution among men (LV – left ventricle; LVH – left ventricular hypertrophy; RWT – relative wall thickness; MMI – myocardial mass index)

After collecting the TTE data, the calculated mean of MMI, RWT, IVSd and PWTd in women were 85.1 ± 26.2 g/m^2^, 0.47 ± 0.10, 10.3 ± 1.9 mm and 10.1 ± 1.8 mm, respectively. The calculated mean of MMI, RWT, IVSd and PWTd in men were 101.5 ± 32.5 g/m^2^, 0.48 ± 0.12, 11.5 ± 1.8 mm and 11.3 ± 1.6 mm, accordingly. 90.6% and 90.6% of subjects with LVH and 19.0% and 14.3% of subjects with normal LV structure had increased IVSd and PWTd, respectively. Based on MMI and RWT, patients were divided into four groups: normal LV structure, LV remodeling, concentric LVH, and eccentric LVH (Figures 1 and 2). The proportion of patients with normal LV structure was 22.1%, with LVH – 33.7% (out of these: 75% – concentric, 25% – eccentric), and with LV remodeling – 44.2%.

### Evaluation of the reliability of ECG criteria in the diagnosis of LVH regardless of gender, age and nutritional status

The sensitivity and specificity of the ECG criteria for the diagnosis of LVH are presented in Table 3. Cornell product and Peguero–Lo Presti criteria had the highest sensitivity, however, Cornell voltage was most specific. RaVL criterion had lowest sensitivity, while Sokolow–Lyon and Peguero–Lo Presti criteria were least specific.

**Table 3.** Sensitivity and specificity of ECG criteria

ECG criterion	Sensitivity (%)	Specificity (%)	AUC	p value (CI = 0.95)
Sokolow–Lyon	9.38	85.71	0.44	p = 0.034
RaVL	6.25	90.48	0.51	p = 0.038
Cornell voltage	21.88	100	0.69	p = 0.084
Cornell product	31.25	95.24	0.72	p = 0.070
Peguero–Lo Presti	31.25	85.71	0.68	p = 0.053

AUC – area under the curve; CI – confidence interval

**Figure 3.**
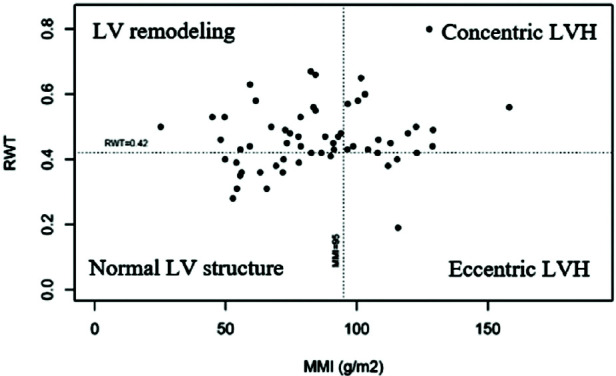
ROC curves of ECG criteria for indication of LVH (light gray dotted line shows the threshold of random diagnostic method)

Based on sensitivity and specificity of the criteria, ROC curves were generated (Figure 3) and AUC of the criteria were calculated (Table 3). After comparing the reference AUC = 1 with the AUC of each criterion it was found that Cornell voltage (Fig. 3 C), Cornell product (Fig. 3 D), and Peguero–Lo Presti (Fig. 3 E) criteria did not differ significantly from the reference AUC (CI = 95%). On the contrary, Sokolow–Lyon (Fig. 3 A) and RaVL (Fig. 3 B) criteria differed from the control group significantly (Table 4). Therefore, the possibility that Cornell voltage, Cornell product and Peguero–Lo Presti criteria may be a reliable method to diagnose LVH cannot be ruled out, unlike Sokolow–Lyon and RaVL criteria. Based on calculated AUCs, Cornell product criterion was the most reliable when diagnosing LVH as opposed to Sokolow–Lyon criterion which was least.

### Evaluation of the reliability of ECG criteria in the diagnosis of LVH depending on gender, age and nutritional status factor

The differences of reliability between all the data of analyzed criteria and the data segregated by gender, age and nutritional status were compared using ANOVA statistical method (Table 5). The results show that there are no statistically significant differences between the mentioned groups (p > 0.05).

## Discussion

The collected general patient data showed that the distribution of patients in gender as well as nutritional status groups in our study was unequal. Nearly twice as many women as men participated in the study, despite the fact that cardiovascular diseases are the leading cause of death among both women and men. Such gender distribution can be related to the fact that in Lithuania in 2012 more women than men were hospitalized with cardiovascular disease, although the duration of hospitalization was similar in both genders [1, 6]. Additionally, preobesity is also known to be a risk factor for cardiovascular diseases [7]. Weight gain particularly increases the risk of arterial hypertension which is most likely to cause the development of LVH [8]. This may be related to the fact that the majority of subjects in the study were overweight or had I–III class obesity. Due to the small number of males and individuals with normal BMI in this study, no strict conclusions could be made regarding the differences in ECG-based LVH diagnosis in these groups.

**Table 4.** The reliability of ECG criteria to diagnose LVH in different gender, age and nutritional status groups

ECG criterion	Factor	Factor groups	p value (CI = 0.95)
Sokolow–Lyon	Gender	Female	0.342
		Male	
	Age (years)	≥ 65	0.312
		< 65	
	BMI (kg/m2)	≥ 30	0.269
		< 30	
RaVL	Gender	Female	0.761
		Male	
	Age (years)	≥ 65	0.523
		< 65	
	BMI (kg/m2)	≥ 30	0.553
		< 30	
Cornell voltage	Age (years)	≥ 65	0.722
		< 65	
	BMI (kg/m2)	≥ 30	0.358
		< 30	
Cornell product	Age (years)	≥ 65	0.079
		< 65	
	BMI (kg/m2)	≥ 30	0.056
		< 30	
Peguero–Lo Presti	Age (years)	≥ 65	0.457
		< 65	
	BMI (kg/m2)	≥ 30	0.325
		< 30	

LVH – left ventricular hypertrophy; BMI – body mass index, kg/m2; CI – confidence interval

In the literature it is seen that patients often have the concentric type of LVH due to the high prevalence of arterial hypertension [9]. As the collected TTE data showed, our study was no exception. IVSd and PWTd were evenly distributed among patients with hypertrophic LVH, indicating the absence of LV wall asymmetry, which could alter ECG results. However, there was an unusually large presentation of patients with eccentric LVH in the study, although this type is described relatively rarely in the literature and is mostly associated with athletes [10]. This phenomenon could be explained in two ways:

1) A part of patients with eccentric LVH had marginal RWT and MMI values possibly due to the onset of LV decompensation. A literature analysis which was conducted in the United Kingdom in 2020 has identified the type of eccentric LVH that develops from the concentric LVH type due to LV expansion at the onset of LV decompensation [11]. This assumption is supported by the fact that the prevalence of heart failure is higher in elderly, especially above the age of 65 years [12]. As mentioned earlier, the majority of subjects in our study were older than 65 years.
2) Eccentric LVH may be a consequence of chronic myocardial ischemia (pre-infarction period) or prior myocardial infarction, as indicated by a study conducted in Lithuania in 2008 [13].

A part of calculated sensitivity and specificity of ECG criteria differed from those that were seen in previous studies. RaVL criterion in our study was found to have lower sensitivity and specificity than was known from the previous studies [14, 15, 16]. Cornell voltage and Cornell product criteria were slightly more reliable for diagnosing LVH and had similar sensitivity and specificity as in other studies [14, 15, 17, 18, 19]. The reliability of Peguero–Lo Presti criterion to diagnose LVH was not as high as described by the developers of this criterion, but the sensitivity and specificity was not as low as in a study conducted in Japan [14, 18]. The sensitivity and specificity of Sokolow–Lyon criterion did not differ from other studies [14, 15, 20, 21, 17].

In this study, the reliability of Sokolow–Lyon and RaVL criteria for diagnosing LVH did not differ between both genders. This contradicts a study conducted in 2015 which found that RaVL criterion was more reliable among women than men [16]. This discrepancy could possibly be explained by the small sample of males in our study.

In this study no statistically significant differences between age groups were observed. Similarly, in a study conducted in 2008 the reliability of Sokolow–Lyon criterion did not differ between different age groups [22]. Nevertheless, a study conducted in 2017 showed that the reliability of Peguero–Lo Presti criterion was higher in those older than 50 years [17]. Meanwhile, in our study, the minimum age of subjects was 48 years, with a mean age of 71 years, thus almost all patients were over 50 years of age. Therefore, statistically significant difference between age groups was not detected.

Our study identified no differences regarding the reliability of ECG criteria among the nutritional status groups. Our results are slightly different from the above-mentioned 2008 study where the sensitivity of Sokolow–Lyon criterion was lower in the obese group compared to the BMI < 30 kg/m^2^ group [22]. The difference in study results may be due to the small sample size of normal BMI subjects in our study. Meanwhile, both studies showed similar reliability for LVH diagnosis of RaVL, Cornell voltage and Cornell product criteria regardless of nutritional status [22].

## Conclusions

Our study showed that Sokolow–Lyon and RaVL criteria were not reliable in LVH diagnosis compared to TTE, while the Cornell voltage, Cornell product, and Peguero–Lo Presti criteria showed to be reliable. Therefore, the latter three criteria show potential to be used in everyday clinical practice. However, further studies with larger sample sizes are needed.

## Study limitations

Due to the fact that often patients with LVH have left or right branch block or paced rhythm in ECG recording, the sample size in our study is relatively small. In addition, there are only five ECG criteria analyzed. We excluded the ECG criteria that are not widely used and also do not have the potential to be used in everyday medical practice because of lower specificity or sensitivity. Furthermore, gender and nutritional status groups in this study were uneven. Therefore, further studies with larger sample sizes, more ECG criteria and even subgroups are needed in order to determine which ECG criterion is most suitable for LVH diagnosis.
